# Supplementary material for: End Sequence Analysis Toolkit (ESAT) expands the extractable information from single-cell RNA-seq data
Source: Genome Res. 2016 Oct;26(10):1397–410. doi: 10.1101/gr.207902.116 (PMC5052061; doi:10.1101/gr.207902.116)
Supplement: Supplemental Material [file supp_gr.207902.116_Supp_Materials.tar.gz › ESAT-esat_v0.1/doc/html/classbroad_1_1pda_1_1gene_1_1_neighbor_analysis.html]

ESAT: broad.pda.gene.NeighborAnalysis Class Reference


ESAT


- Main Page
- Related Pages
- Packages
- Classes
- Files

- Class List
- Class Index
- Class Hierarchy
- Class Members

All Classes Namespaces Files Functions Variables Enumerator Pages

- broad
- pda
- gene
- NeighborAnalysis

Public Member Functions |
Static Public Member Functions |
List of all members

broad.pda.gene.NeighborAnalysis Class Reference

|  |  |
| --- | --- |
| Public Member Functions | |
|  | NeighborAnalysis () |
|  | |
|  | NeighborAnalysis (String setname, String refname) throws IOException |
|  | |
|  | NeighborAnalysis (BEDFileParser newGeneSet, BEDFileParser newRefSet) throws IOException |
|  | |
| void | getNeighbors (BEDFileParser neighborsBed, BufferedWriter bw, boolean toWrite, double distThreshold) throws IOException |
|  | |
| void | updateDistanceToNeighbors () throws IOException |
|  | |
| GeneWithIsoforms[] | getNeighbors (Gene g) throws IOException |
|  | |
| double[] | getNeighborDistance (Gene g) throws IOException |
|  | |

|  |  |
| --- | --- |
| Static Public Member Functions | |
| static void | main (String[] args) throws Exception |
|  | |
| static GeneWithIsoforms | getLeftNeighbor (Gene g, BEDFileParser ref) |
|  | |
| static GeneWithIsoforms | getRightNeighbor (Gene g, BEDFileParser ref) |
|  | |

## Constructor & Destructor Documentation

|  |  |  |  |  |
| --- | --- | --- | --- | --- |
| broad.pda.gene.NeighborAnalysis.NeighborAnalysis | ( |  | ) |  |

Here is the caller graph for this function:

|  |  |  |  |
| --- | --- | --- | --- |
| broad.pda.gene.NeighborAnalysis.NeighborAnalysis | ( | String | *setname*, |
|  |  | String | *refname* |
|  | ) |  | throws IOException |

|  |  |  |  |
| --- | --- | --- | --- |
| broad.pda.gene.NeighborAnalysis.NeighborAnalysis | ( | BEDFileParser | *newGeneSet*, |
|  |  | BEDFileParser | *newRefSet* |
|  | ) |  | throws IOException |

## Member Function Documentation

|  |  |  |  |
| --- | --- | --- | --- |
| static GeneWithIsoforms broad.pda.gene.NeighborAnalysis.getLeftNeighbor | ( | Gene | *g*, |
|  |  | BEDFileParser | *ref* |
|  | ) |  |  |

static

Here is the call graph for this function:

Here is the caller graph for this function:

|  |  |  |  |  |  |
| --- | --- | --- | --- | --- | --- |
| double [] broad.pda.gene.NeighborAnalysis.getNeighborDistance | ( | Gene | *g* | ) | throws IOException |

Here is the call graph for this function:

|  |  |  |  |
| --- | --- | --- | --- |
| void broad.pda.gene.NeighborAnalysis.getNeighbors | ( | BEDFileParser | *neighborsBed*, |
|  |  | BufferedWriter | *bw*, |
|  |  | boolean | *toWrite*, |
|  |  | double | *distThreshold* |
|  | ) |  | throws IOException |

Here is the call graph for this function:

Here is the caller graph for this function:

|  |  |  |  |  |  |
| --- | --- | --- | --- | --- | --- |
| GeneWithIsoforms [] broad.pda.gene.NeighborAnalysis.getNeighbors | ( | Gene | *g* | ) | throws IOException |

Here is the call graph for this function:

|  |  |  |  |
| --- | --- | --- | --- |
| static GeneWithIsoforms broad.pda.gene.NeighborAnalysis.getRightNeighbor | ( | Gene | *g*, |
|  |  | BEDFileParser | *ref* |
|  | ) |  |  |

static

Here is the call graph for this function:

Here is the caller graph for this function:

|  |  |  |  |  |  |
| --- | --- | --- | --- | --- | --- |
| static void broad.pda.gene.NeighborAnalysis.main | ( | String[] | *args* | ) | throws Exception |

static

Here is the call graph for this function:

|  |  |  |  |  |
| --- | --- | --- | --- | --- |
| void broad.pda.gene.NeighborAnalysis.updateDistanceToNeighbors | ( |  | ) | throws IOException |

Here is the call graph for this function:

Here is the caller graph for this function:

---

The documentation for this class was generated from the following file:

- java/broad/pda/gene/NeighborAnalysis.java


---

Generated on Fri Aug 15 2014 15:11:00 for ESAT by  

 1.8.7
